# Inflammation, glucose metabolism, and nutritional markers in relation to all-cause and cardiac mortality among initial hemodialysis patients: a multicenter cohort study

**DOI:** 10.3389/fnut.2025.1660267

**Published:** 2025-11-06

**Authors:** Shi-mei Hou, Yu-ting Gao, Meng-huan Wu, Yu-xin Ren, Jing Zheng, Yao Wang, Jing-yuan Cao, Xiao-xu Wang, Yan Yang, Bin Wang, Min Yang, Min Li

**Affiliations:** 1 Department of Nephrology, the Third Affiliated Hospital of Soochow University, Changzhou First People’s Hospital, Changzhou, China; 2 School of Medicine, Zhong Da Hospital, Institute of Geriatrics, Southeast University, Nanjing, Jiangsu, China; 3 Institute of Nephrology, Yangzhou First People’s Hospital, Yangzhou, Jiangsu, China; 4 School of Medicine, Zhong Da Hospital, Institute of Nephrology, Southeast University, Nanjing, Jiangsu, China

**Keywords:** inflammation, glucose metabolism, nutrition, biomarkers, mortality, hemodialysis

## Abstract

**Objective:**

To investigate the prognostic value of inflammatory biomarkers including neutrophil-to-lymphocyte ratio (NLR), platelet-to-lymphocyte ratio (PLR), and lymphocyte-to-monocyte ratio (LMR), glucose metabolism (glucose-to-lymphocyte ratio, GLR), and nutritional (albumin, ALB) biomarkers for predicting all-cause and cardiac mortality in patients initiating hemodialysis (HD), and evaluates their incremental value when integrated into traditional risk models.

**Methods:**

A retrospective cohort of 795 initial HD patients (2014–2020) was analyzed, with follow-up through 2022. Cox proportional hazards models were used to assess associations between biomarkers and mortality. Predictive performance was evaluated using time-dependent ROC curves, C-index, net reclassification improvement (NRI), and integrated discrimination improvement (IDI). Patients were randomly assigned to training (*n* = 557) and validation (*n* = 238) sets, and a survival nomogram was developed based on a full-risk model incorporating both traditional and biomarker variables.

**Results:**

Elevated NLR, PLR, and GLR were independently associated with increased all-cause and cardiac mortality, whereas lower LMR and ALB were protective (all *p* < 0.05). NLR exhibited the highest predictive accuracy across 1-, 3-, and 5-year intervals, followed by GLR and PLR. The full-risk model significantly outperformed the baseline model, with AUCs up to 0.980 and 0.966 for all-cause mortality and 0.947 and 0.978 for cardiac mortality in training and validation sets, respectively (all *p* < 0.001). Improvements in C-index, NRI, and IDI supported its enhanced predictive utility.

**Conclusion:**

Incorporating inflammatory, glucose metabolism and nutritional biomarkers into traditional risk models substantially improves long-term mortality risk stratification in initial HD patients, offering a robust, clinically applicable tool to support individualized prognostic assessment and intervention planning.

## Graphical abstract

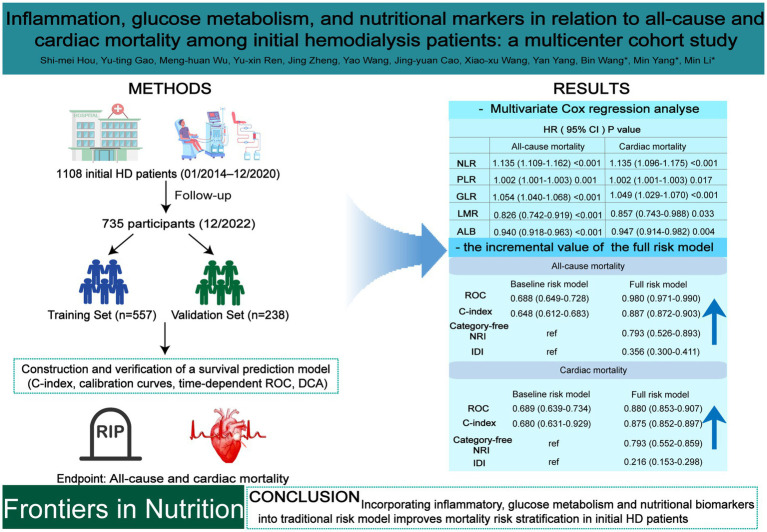

## 1 Introduction

End-stage kidney disease (ESKD) is a critical global public health challenge. By 2023, approximately 10.45 million patients worldwide required renal replacement therapy (RRT) for ESKD (1), with most undergoing hemodialysis (HD). HD patients face significantly elevated mortality rates compared to the general population, primarily due to cardiovascular complications (2), underscoring the need for early risk factor identification.

Beyond traditional risk factors like age, diabetes, and coronary heart disease (CHD), emerging biomarkers are associated with survival in HD patients. Chronic kidney disease (CKD) patients exhibit persistent low-grade inflammation driven by uremic toxins, oxidative stress, gut dysbiosis, and adipose tissue changes (3). At the molecular level, chronic inflammatory states in ESRD are regulated by complex signaling pathways. The nuclear factor κB (NF-κB) pathway is a key proinflammatory pathway that can be activated by uremia toxins and oxidative stress, promote neutrophil production, release and delay neutrophils’ apoptosis, leading to an increase in neutrophil count (4). On the other hand, lymphopenia and dysfunction are at the heart of ESRD immunodeficiency. Adenosine monophosphate activated protein kinase (AMPK) plays an important role in regulating T cell fate as a cellular energy sensor. Studies have shown that AMPK activation can promote the differentiation and function of regulatory T cells (Tregs) while inhibiting the proinflammatory response of helper T cells 17 (5). In the pathological environment of ESRD, AMPK signaling pathway may be impaired, thereby aggravating lymphocyte apoptosis and loss of anti-inflammatory function, resulting in decreased lymphocyte count and abnormal function (6). Therefore, the inflammatory ratios such as NLR may reflect the inherent imbalance between NF-κB-driven proinflammatory forces and AMPK-related anti-inflammatory forces from a macroscopic perspective. This inflammation intensifies with declining renal function and increases risks of cardiovascular events and mortality in dialysis patients (7).

Neutrophil-to-lymphocyte ratio (NLR), platelet-to-lymphocyte ratio (PLR), and lymphocyte-to-monocyte ratio (LMR) are simple, cost-effective inflammatory biomarkers (8) linked to prognosis in conditions like malignancies and coronary artery disease (9–14). They also show predictive value in non-dialysis CKD and peritoneal dialysis (PD) populations (15–17). However, their role in predicting HD mortality is inconsistent. Some studies link NLR and PLR to all-cause mortality, with only PLR being an independent predictor (18). Others found NLR superior to PLR (19), or suggest LMR—but not NLR—predicts mortality in HD (20).

The glucose-to-lymphocyte ratio (GLR), reflecting systemic glucose metabolism and inflammation, has prognostic significance in conditions like COPD exacerbations and malignancies (21–23). Zhong et al. (24) showed GLR outperformed NLR, PLR, and LMR in predicting pancreatic cancer survival. Elevated GLR is also associated with increased death risk in PD patients (25), but its relationship with HD mortality remains unexplored.

Malnutrition, driven by metabolic acidosis, inadequate intake, and nutrient loss during dialysis, further increases mortality risk in HD. Serum albumin (ALB), a key nutritional biomarker, consistently predicts mortality.

This study comprehensively evaluates the relationships between nutritional (ALB), inflammatory (NLR, PLR, LMR), and glucose metabolism (GLR) biomarkers and the risks of all-cause and cardiac mortality in an initial HD cohort. We also explore whether integrating these biomarkers with traditional risk factors enhances mortality risk stratification accuracy.

## 2 Methods

### 2.1 Patients

This multicenter retrospective cohort study enrolled initial HD patients aged 18 to 75 years treated between January 2014 and December 2020 at the nephrology departments of the Third Affiliated Hospital of Soochow University, Zhongda Hospital Affiliated to Southeast University, Taizhou First People’s Hospital, and Yangzhou First People’s Hospital. Patients were excluded if they met any of the following criteria: (1) missing data on neutrophils, lymphocytes, monocytes, serum albumin, platelets, or fasting glucose; (2) history of kidney transplantation (KT) with no further need for HD; (3) presence of comorbid liver diseases (e.g., hepatitis, cirrhosis), hematological disorders (e.g., leukemia, lymphoma), or autoimmune diseases (e.g., systemic lupus erythematosus); (4) malignancy; (5) major surgery or severe trauma (e.g., fractures, burns) within the previous 3 months; (6); documented infections (e.g., pneumonia, sepsis) within the past month. A total of 36 patients (11.5%) were excluded from this study due to missing critical data. No significant differences were observed between the excluded patients and the finally included cohort regarding gender, age, BMI, smoking history, medical history (diabetes, hypertension, CHD), medication history [angiotensin-converting enzyme inhibitor (ACEI), angiotensin receptor blockers (ARB), *β*-blockers], all-cause mortality, or cardiac mortality (all *p* > 0.05; Supplementary Table 1). Detailed information regarding missing data is provided in the Supplementary materials.

The study protocol was approved by the Ethics Committee of the Third Affiliated Hospital of Soochow University (Approval ID: 2024CL085-01) and registered with the Chinese Clinical Trial Registry (ChiCTR 2,300,068,453; registration date: The date of registration was 2023-02-20). All procedures complied with the Declaration of Helsinki and relevant regulatory guidelines.

### 2.2 Clinical covariates

Patient data were extracted from electronic medical records (EMRs), including age, sex, height, weight, smoking history, comorbidities [diabetes, hypertension, coronary heart disease (CHD)], and medication use (ACEI, ARB, *β*-blockers, and lipid-lowering agents). Fasting venous blood samples were collected before dialysis during the long interdialytic interval. Laboratory parameters including hemoglobin (Hb), platelet count (PLT), serum albumin (ALB), absolute neutrophil count (N), absolute lymphocyte count (L), absolute monocyte count (M), fasting blood glucose (FBG), serum creatinine (SCr), and cystatin C (CysC) were measured using a UniCelDxC 800 automated biochemical analyzer (Beckman Coulter, United States). All baseline data were independently reviewed and validated by two senior clinicians. Refer to the Supplementary material for relevant index calculations.

### 2.3 Outcome events

Patients were prospectively followed according to a standardized protocol using outpatient and inpatient medical record systems, as well as telephone interviews, until the occurrence of an endpoint event or the termination date of the study (December 31, 2022). Endpoint events included all-cause mortality and cardiac mortality, with the latter defined as death directly attributable to cardiac conditions or where cardiac disease was the primary contributing factor. Senior resident physicians who documented the time and cause of death were blinded to all other study outcomes.

### 2.4 Statistical analyses

Normality of continuous variables was assessed using the Shapiro–Wilk test. Normally distributed data are presented as mean ± standard deviation (SD); non-normal data as median (interquartile range, IQR). Categorical variables are reported as counts (percentages). Group comparisons used Pearson *χ*^2^ or Fisher’s exact test for categorical variables, and t-tests or Wilcoxon rank-sum tests for continuous variables. Spearman correlation analysis assessed associations among NLR, GLR, PLR, LMR, and ALB. Univariate Cox regression analysis was employed to explore the relationship between all-cause and cardiac mortality and the following variables: sex, age, BMI, smoking history. Comorbidities (diabetes, hypertension and CHD), medication use (*β*-blockers, ACEI/ARB and lipid-lowering agents), and laboratory parameters (Hb, NLR, PLR, GLR, LMR, ALB and SCr/CysC). Variables with statistical significance (*p* < 0.05) in univariate analyses were included in multivariate Cox regression models to identify the independent risk or protective factors. The variance expansion factor (VIF) values for all variables range from 1 to 2, indicating no significant multicollinearity in the multivariable models. Time-dependent receiver operating characteristic (ROC) curves were generated to compare the predictive performance of NLR, PLR, GLR, LMR, and ALB for 1-, 3-, and 5-year mortality using the area under the curve (AUC) and DeLong’s test. Two prognostic models were developed: a baseline risk model based on traditional factors (age and comorbidities), and a full model integrating traditional risk factors with biomarkers of nutrition (ALB), inflammation (NLR, PLR, LMR) and glucose metabolism (GLR). Model performance was evaluated using AUC, concordance index (C-index), category-free net reclassification improvement (NRI) and integrated discrimination improvement (IDI).

The cohort was randomly divided into a training set (*n* = 557) and an internal validation set (*n* = 238) at a 7:3 ratio. The construction and validation of the survival nomogram are detailed in the Supplementary material.

Statistical significance was defined as a two-tailed *p* values < 0.05. All analyses were performed using IBM SPSS Statistics for Windows, version 26 (IBM Corp, Armonk, NY, United States), and R software.^1^

## 3 Results

### 3.1 Baseline characteristics

**Figure 1:**
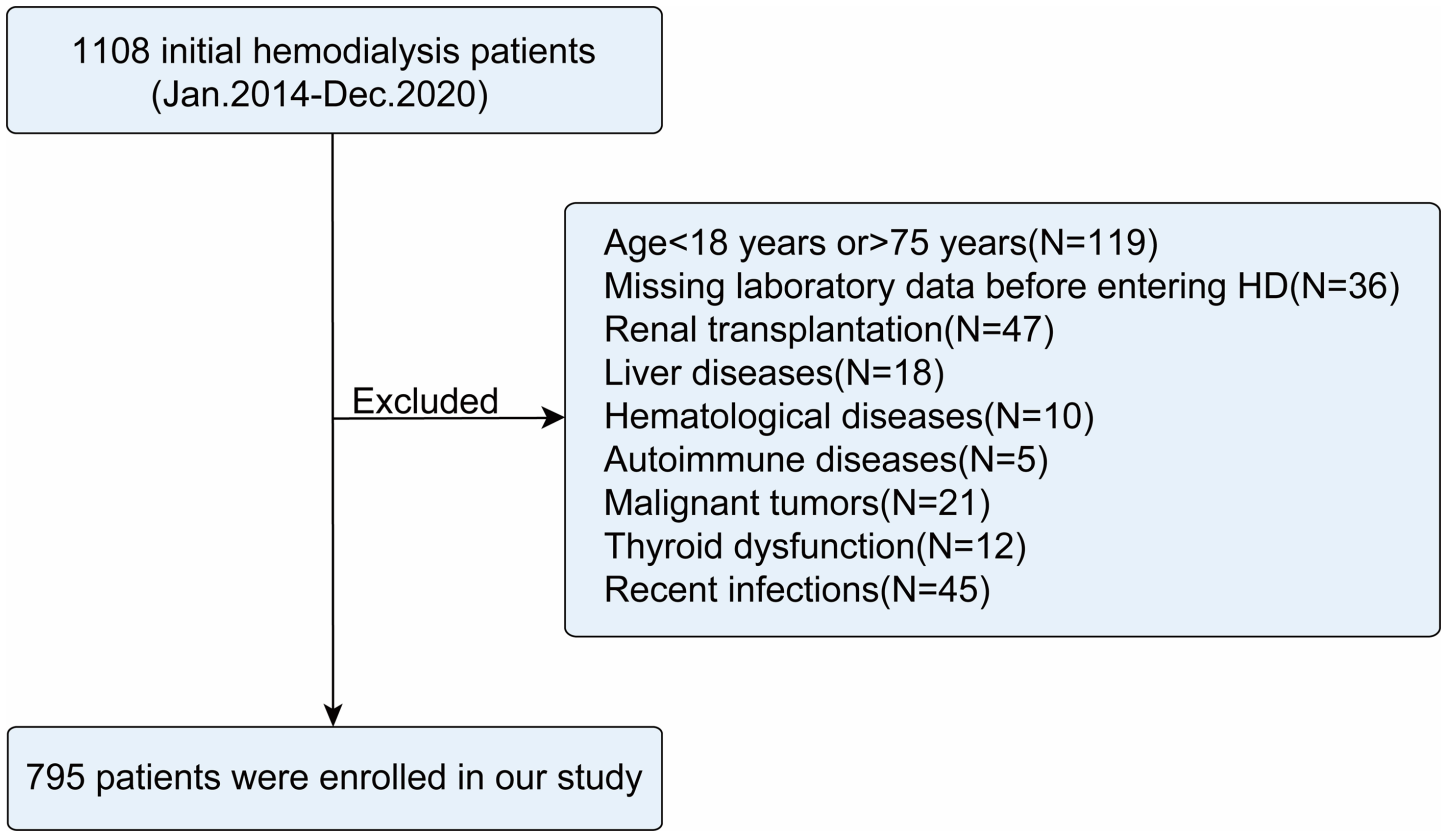
Flow chart of the study.

A total of 795 initial HD patients were included in this study (Figure 1), with the majority aged 18–65 years (76.48%) and male (64.15%). The median BMI was 23.4 kg/m^2^. During the follow-up period, 239 patients (30.06%) died, of whom 114 (47.70%) succumbed to cardiac causes (Table 1).

**Table 1:** Demographics and baseline characteristics of the study.

Characteristics	Overall (*n* = 795)	All-cause mortality			Cardiac mortality		
		Alive (*n* = 556)	Dead (*n* = 239)	*p* -value	Alive (*n* = 681)	Dead (*n* = 114)	*p* -value
Sex, *n* (%)				0.913			0.855
Female	285 (35.85%)	200 (35.97%)	85 (35.56%)		245 (35.98%)	40 (35.09%)	
Male	510 (64.15%)	356 (64.03%)	154 (64.44%)		436 (64.02%)	74 (64.91%)	
Age, year, *n* (%)				**<0.001**			**<0.001**
18–65 years	608 (76.48%)	468 (84.17%)	140 (58.58%)		542 (79.59%)	66 (57.89%)	
66–75 years	187 (23.52%)	88 (15.83%)	99 (41.42%)		139 (20.41%)	48 (42.11%)	
Heigh, m, median (IQR)	1.68 (1.60, 1.72)	1.68 (1.60, 1.72)	1.66 (1.60, 1.72)	**0.049**	1.68 (1.60, 1.72)	1.67 (1.60, 1.72)	0.486
Weight, kg, median (IQR)	65 (57, 74)	65 (57, 75)	65 (57, 72)	**0.202**	65 (57, 74)	65 (58, 73)	0.867
BMI, kg/m2, median (IQR)	23.40 (21.10, 26.20)	23.40 (21.10, 26.20)	23.40 (21.10, 26.30)	0.747	23.40 (21.10, 26.10)	23.90 (21.20, 26.60)	0.378
Smoking history, *n* (%)	172 (21.64%)	108 (19.42%)	64 (26.78%)	**0.021**	140 (20.56%)	32 (28.07%)	0.071
Comorbidities, *n* (%)							
CHD	104 (13.08%)	57 (10.25%)	47 (19.67%)	**<0.001**	71 (10.43%)	33 (28.95%)	**<0.001**
Hypertension	727 (91.45%)	509 (91.55%)	218 (91.21%)	0.878	622 (91.34%)	105 (92.11%)	0.786
Diabetes	352 (44.28%)	213 (38.31%)	139 (58.16%)	**<0.001**	280 (41.12%)	72 (63.16%)	**<0.001**
Medication history, *n* (%)							
ACEI/ARB	194 (24.40%)	129 (23.20%)	65 (27.20%)	0.229	162 (23.79%)	32 (28.07%)	0.325
β-blocker	478 (60.13%)	354 (63.67%)	124 (51.88%)	**0.002**	410 (60.21%)	68 (59.65%)	0.911
Lipid lowing agent	170 (21.38%)	99 (17.81%)	71 (29.71%)	**<0.001**	128 (18.80%)	42 (36.84%)	**<0.001**
Laboratory data							
Hb, g/L, median (IQR)	81 (71, 93)	80 (70, 92)	83 (74, 96)	**0.018**	80 (71, 93)	83 (75, 98)	**0.012**
NLR, median (IQR)	5.60 (3.70, 8.60)	4.3 (3.30, 5.90)	13.40 (8.80, 16.00)	**<0.001**	5.00 (3.50, 7.20)	13.40 (8.70, 15.80)	**<0.001**
PLR, median (IQR)	148 (109, 196)	133 (100, 168)	196 (150, 274)	**<0.001**	140 (105, 185)	187 (149, 259)	**<0.001**
GLR, median (IQR)	9 (6, 15)	7 (5, 10)	18 (13, 25)	**<0.001**	8 (5, 14)	18 (12, 25)	**<0.001**
LMR, median (IQR)	3.33 (2.38, 4.59)	3.70 (2.78, 5.00)	2.63 (1.96, 3.69)	**<0.001**	3.45 (2.44, 4.76)	2.78 (2.22, 3.70)	**<0.001**
ALB, median (IQR)	33.20 (30.10, 36.60)	34.3 (31.30, 37.20)	30.80 (27.50, 34.00)	**<0.001**	33.70 (30.60, 36.80)	30.90 (27.50, 34.20)	**<0.001**
SCr/CysC, median (IQR)	22 (15, 30)	22 (16, 30)	23 (14, 30)	0.741	23 (15, 30)	22 (14, 30)	0.896

BMI, body mass index; CHD, coronary heart disease; ACEI, angiotensin-converting enzyme inhibitor; ARB, angiotensin receptor blocker; IQR, interquartile range; Hb, hemoglobin; NLR, neutrophil to lymphocyte ratio; PLR, platelet to lymphocyte ratio; GLR, glucose to lymphocyte ratio; LMR, lymphocyte to monocyte ratio; ALB, albumin; SCr/CysC, ratio of serum creatinine to cystatin C. Bold values indicates significant differences in characteristics between groups of initial hemodialysis patients who survived and died.

Compared to the survival group, both the all-cause and cardiac mortality groups had significantly higher proportions of elderly patients (15.83% *vs.* 41.42 and 20.41% *vs.* 42.11%, respectively), users of lipid-lowering agents (17.81% *vs.* 29.71 and 18.80% *vs.* 36.84%), and patients with diabetes (38.31% *vs.* 58.16 and 41.12% *vs.* 63.16%) or CHD (10.25% *vs.* 19.67 and 10.43% *vs.* 28.95%; all *p* < 0.001). Additionally, the all-cause and cardiac mortality groups exhibited higher levels of Hb (83 *vs.* 80 for both), NLR (13.40 *vs.* 4.30 and 13.40 *vs.* 5.00), PLR (196 *vs.* 133 and 187 *vs.* 140), and GLR (18 *vs.* 7 and 18 *vs.* 8) (all *p* < 0.05), as well as significantly lower LMR (2.63 *vs.* 3.70 and 2.78 *vs.* 3.45) and ALB (30.80 *vs.* 34.30 and 30.90 *vs.* 33.70) (all *p* < 0.001). No significant differences were observed between the groups in terms of sex, BMI, hypertension, use of ACEI, or SCr/CysC levels (all *p* > 0.05).

### 3.2 Correlation analyses of inflammation, glucose metabolism, and nutrition markers

See Supplementary Figure 1.

### 3.3 Relationship between inflammation, glucose metabolism, nutrition markers and mortality outcomes

#### 3.3.1 Univariate and multivariate cox regression analyses

As shown in Table 2, univariate Cox regression analysis identified age, comorbid diabetes and CHD, use of lipid-regulating agents, higher levels of Hb, NLR, PLR, GLR, and SCr/CysC, along with lower levels of LMR and ALB, as risk factors for all-cause mortality (all *p* < 0.05). The use of *β*-blockers was identified as a protective factor (*p* < 0.05). Variables with statistical significance in the univariate analyses were included in the multivariate Cox regression analyses. The results demonstrated that age [HR (95% CI): 1.695 (1.294–2.221), *p* < 0.001], higher NLR [HR (95% CI): 1.135 (1.109–1.162), *p* < 0.001], PLR [HR (95% CI): 1.002 (1.001–1.003), *p* = 0.001], GLR [HR (95% CI): 1.054 (1.040–1.068), *p* < 0.001], as well as lower LMR [HR (95% CI): 0.826 (0.742–0.919), *p* < 0.001] and ALB [HR (95% CI): 0.940 (0.918–0.963), *p* < 0.001], were independent risk factors for all-cause mortality.

**Table 2:** Univariate and multivariate Cox regression analyses for all-cause mortality.

Characteristics	Univariate Cox regression analyses		Multivariate Cox regression analyses	
	HR (95% CI)	*p*-value	HR (95% CI)	*p*-value
Sex (Male)	0.991 (0.760, 1.292)	0.947		
Age (66–75 years)	2.869 (2.214, 3.718)	**<0.001**	1.695 (1.294, 2.221)	**<0.001**
BMI, kg/m^2^	0.984 (0.954, 1.016)	0.336		
Smoking history	1.267 (0.951, 1.688)	0.106		
Diabetes	1.918 (1.483, 2.481)	**<0.001**		
Hypertension	0.904 (0.577, 1.414)	0.658		
CHD	1.823 (1.325, 2.509)	**<0.001**		
Hb, g/L	1.007 (1.000, 1.013)	**0.046**		
NLR	1.216 (1.194, 1.239)	**<0.001**	1.135 (1.109, 1.162)	**<0.001**
PLR	1.005 (1.004, 1.005)	**<0.001**	1.002 (1.001, 1.003)	**0.001**
GLR	1.106 (1.093, 1.119)	**<0.001**	1.054 (1.040, 1.068)	**<0.001**
LMR	0.615 (0.551, 0.687)	**<0.001**	0.826 (0.742, 0.919)	**<0.001**
ALB, g/L	0.903 (0.883, 0.923)	**<0.001**	0.940 (0.918, 0.963)	**<0.001**
β-blocker	0.637 (0.494, 0.822)	**<0.001**		
ACEI/ARB	1.161 (0.873, 1.544)	0.305		
Lipid lowing agents	1.688 (1.279, 2.230)	**<0.001**		
SCr/CysC	1.003 (1.001, 1.005)	**0.002**		

HR, hazard ratio; CI, confidence Interval; BMI, body mass index; CHD, coronary heart disease; Hb, hemoglobin; NLR, neutrophil to lymphocyte ratio; PLR, platelet to lymphocyte ratio; GLR, glucose to lymphocyte ratio; LMR, lymphocyte to monocyte ratio; ALB, albumin; ACEI, angiotensin-converting enzyme inhibitor; ARB, angiotensin receptor blocker; SCr/CysC, ratio of serum creatinine to cystatin C. Bold values corresponding feature is significantly associated with all-cause mortality.

As presented in Table 3, the risk factors for cardiac mortality were largely consistent with those for all-cause mortality, with the exception of SCr/CysC (all *p* < 0.05). Multivariate Cox regression analyses revealed that advanced age [HR (95% CI): 1.736 (1.176–2.564), *p* = 0.006], comorbid CHD [HR (95% CI): 1.555 (1.007–2.401), *p* = 0.046], higher NLR [HR (95% CI): 1.135 (1.096–1.175), *p* < 0.001], PLR [HR (95% CI): 1.002 (1.001–1.003), *p* = 0.017], GLR [HR (95% CI): 1.049 (1.029–1.070), *p* < 0.001], as well as lower LMR [HR (95% CI): 0.857 (0.743–0.988), *p* = 0.033] and ALB [HR (95% CI): 0.947 (0.914–0.982), *p* = 0.004], were independent risk factors for cardiac mortality.

**Table 3:** Univariate and multivariate Cox regression analyses for cardiac mortality.

Characteristics	Univariate Cox regression analyses		Multivariate Cox regression analyses	
	HR (95% CI)	*p*-value	HR (95% CI)	*p*-value
Sex (Male)	1.012 (0.689, 1,487)	0.950		
Age (66–75 years)	2.967 (2.042, 4.313)	**<0.001**	1.736 (1.176, 2.564)	**0.006**
BMI, kg/m^2^	1.005 (0.961, 1.051)	0.837		
Smoking history	1.348 (0.896, 2.030)	0.152		
Diabetes	2.376 (1.623, 3.477)	**<0.001**		
Hypertension	1.004 (0.508, 1.984)	0.990		
CHD	3.074 (2.049, 4.61)1	**<0.001**	1.555 (1.007, 2.401)	**0.046**
Hb, g/L	1.011 (1.001, 1.020)	**0.025**		
NLR	1.215 (1.183, 1.247)	**<0.001**	1.135 (1.096, 1.175)	**<0.001**
PLR	1.004 (1.003, 1.005)	**<0.001**	1.002 (1.001, 1.003)	**0.017**
GLR	1.103 (1.085, 1.122)	**<0.001**	1.049 (1.029, 1.070)	**<0.001**
LMR	0.704 (0.608, 0.815)	**<0.001**	0.857 (0.743, 0.988)	**0.033**
ALB, g/L	0.910 (0.881, 0.940)	**<0.001**	0.947 (0.914, 0.982)	**0.004**
β-blocker	0.873 (0.600, 1.269)	0.476		
ACEI/ARB	1.220 (0.811, 1.836)	0.340		
Lipid lowing agents	2.351 (1.605, 3.444)	**<0.001**		
SCr/CysC	1.003 (1.000, 1.006)	0.072		

HR, hazard ratio; CI, confidence Interval; BMI, body mass index; CHD, coronary heart disease; Hb, hemoglobin; NLR, neutrophil to lymphocyte ratio; PLR, platelet to lymphocyte ratio; GLR, glucose to lymphocyte ratio; LMR, lymphocyte to monocyte ratio; ALB, albumin; ACEI, angiotensin-converting enzyme inhibitor; ARB, angiotensin receptor blocker; SCr/CysC, ratio of serum creatinine to cystatin C. Bold values corresponding feature is significantly associated with all-cause mortality.

#### 3.3.2 Time-dependent ROC curve analysis

**Figure 2:**
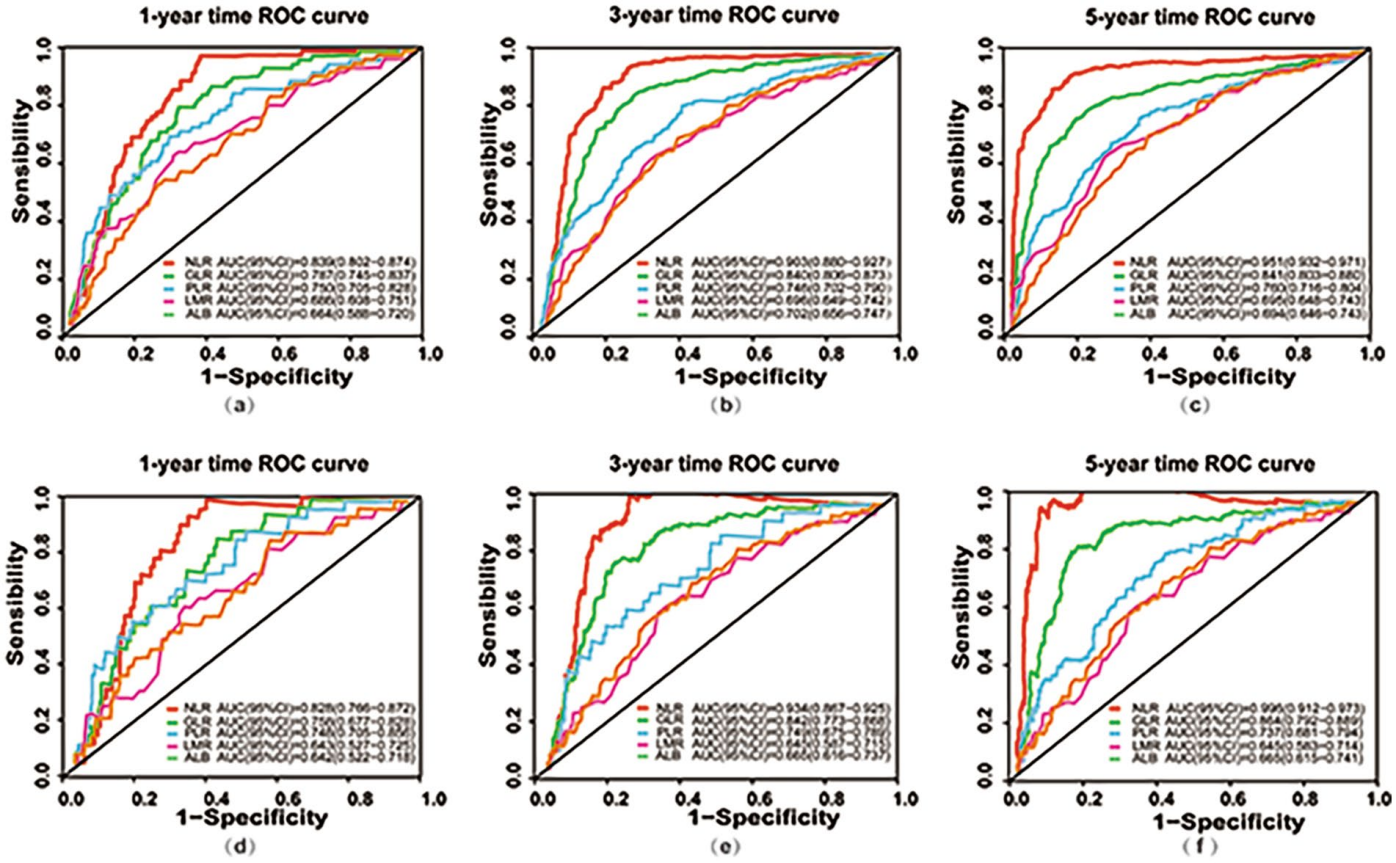
Time-dependent ROC curves for predicting all-cause and cardiac mortality using nutritional, inflammatory, and glucose metabolism markers. **(a–c)** ROC curves at 1-year **(a)**, 3-year **(b)**, and 5-year **(c)** for predicting all-cause mortality based on nutritional (ALB), inflammatory (NLR, PLR, LMR), and glucose metabolism (GLR) markers; **(d–f)** ROC curves at 1-year **(d)**, 3-year **(e)**, and 5-year **(f)** for predicting cardiac mortality using nutritional (ALB), inflammatory (NLR, PLR, LMR), and glucose metabolism (GLR) markers. ROC, receiver operating characteristic; AUC, area under the curve; CI, Confidence Interval; NLR, neutrophil to lymphocyte ratio; PLR, platelet to lymphocyte ratio; GLR, glucose to lymphocyte ratio; LMR, lymphocyte to monocyte ratio; ALB, albumin.

Time-dependent ROC curves was employed to evaluate the predictive performance of nutritional, inflammatory, and glucose metabolism markers for all-cause and cardiac mortality. As shown in Figure 2 and detailed in Tables 4, 5, the inflammatory marker NLR exhibited the highest predictive accuracy at 1, 3, and 5 years for all-cause and cardiac mortality. The AUC and corresponding 95% CI for all-cause mortality were 0.839 (0.802–0.874), 0.903 (0.880–0.927) and 0.951 (0.932–0.971), respectively. For cardiac mortality, the AUC values were 0.828 (0.766–0.872), 0.934 (0.867–0.925), and 0.996 (0.912–0.973) (all *p* < 0.001). The glucose metabolism marker GLR ranked second in predictive value, with AUC (95% CI) values of 0.787 (0.745–0.837), 0.840 (0.806–0.873), 0.841 (0.803–0.880) for all-cause mortality and 0.758 (0.677–0.828), 0.842 (0.773–0.868), 0.864 (0.792–0.889) for cardiac mortality. In contrast, the inflammatory marker LMR and the nutritional marker ALB showed comparatively lower predictive ability for both outcomes. There was no statistically significant difference in predictive value between these two markers (all *p* > 0.05).

**Table 4:** Predictive value of nutritional, inflammatory, and glucose metabolic markers for all-cause mortality at 1, 3, and 5 years.

1-year time-dependent ROC					
^Index^AUC (95% CI)	^Index^AUC (95% CI)				
	^NLR^0.839 (0.802–0.874)	^GLR^0.787 (0.745–0.837)	^PLR^0.750 (0.705–0.828)	^LMR^0.686 (0.608–0.751)	^ALB^0.664 (0.588–0.720)
^NLR^0.839 (0.802–0.874)	–	*p* < 0.001	*p* < 0.001	*p* < 0.001	*p* < 0.001
^GLR^0.787 (0.745–0.837)	***	–	0.660	*p* < 0.001	*p* < 0.001
^PLR^0.750 (0.705–0.828)	***	0.660	–	*p* < 0.001	*p* < 0.001
^LMR^0.686 (0.608–0.751)	***	***	***	–	0.551
^ALB^0.664 (0.588–0.720)	***	***	***	0.551	–

3-year time-dependent ROC					
^Index^AUC (95% CI)	^Index^AUC (95% CI)				
	^NLR^0.903 (0.880–0.927)	^GLR^0.840 (0.806–0.873)	^PLR^0.746 (0.702–0.790)	^LMR^0.696 (0.649–0.742)	^ALB^0.702 (0.656–0.747)
^NLR^0.903 (0.880–0.927)	–	*p* < 0.001	*p* < 0.001	*p* < 0.001	*p* < 0.001
^GLR^0.840 (0.806–0.873)	***	–	*p* = 0.002	*p* < 0.001	*p* < 0.001
^PLR^0.746 (0.702–0.790)	***	*p* = 0.002	–	*p* < 0.001	*P* < 0.001
^LMR^0.696 (0.649–0.742)	***	***	***	–	0.851
^ALB^0.702 (0.656–0.747)	***	***	***	0.851	–

5-year time-dependent ROC					
^Index^AUC (95% CI)	^Index^AUC (95% CI)				
	^NLR^0.951 (0.932–0.971)	^GLR^0.841 (0.803–0.880)	^PLR^0.760 (0.716–0.804)	^LMR^0.695 (0.648–0.743)	^ALB^0.694 (0.646–0.743)
^NLR^0.951 (0.932–0.971)	–	*p* < 0.001	*p* < 0.001	*p* < 0.001	*p* < 0.001
^GLR^0.841 (0.803–0.880)	***	–	*p* = 0.006	*p* < 0.001	*p* < 0.001
^PLR^0.760 (0.716–0.804)	***	*p* = 0.006	–	*p* < 0.001	*p* < 0.001
^LMR^0.695 (0.648–0.743)	***	***	***	–	0.977
^ALB^0.694 (0.646–0.743)	***	***	***	0.977	–

ROC, receiver operating characteristic; AUC, area under the curve; CI, confidence Interval; NLR, neutrophil to lymphocyte ratio; PLR, platelet to lymphocyte ratio; GLR, glucose to lymphocyte ratio; LMR, lymphocyte to monocyte ratio; ALB, albumin. *, *p* < 0.05; **, *p* < 0.01; ***, *p* < 0.001.

**Table 5:** Predictive value of nutritional, inflammatory, and glucose metabolic markers for cardiac mortality at 1, 3, and 5 years.

1-year time-dependent ROC					
^Index^AUC (95% CI)	^Index^AUC (95% CI)				
	^NLR^0.828 (0.766–0.872)	^GLR^0.758 (0.677–0.828)	^PLR^0.748 (0.705–0.856)	^LMR^0.643 (0.527–0.725)	^ALB^0.642 (0.522–0.718)
^NLR^0.828 (0.766–0.872)	–	*p* < 0.001	*p* < 0.001	*p* < 0.001	*p* < 0.001
^GLR^0.758 (0.677–0.828)	***	–	0.644	*p* < 0.001	*p* < 0.001
^PLR^0.748 (0.705–0.856)	***	0.644	–	*p* < 0.001	*p* < 0.001
^LMR^0.643 (0.527–0.725)	***	***	***	–	0.917
^ALB^0.642 (0.522–0.718)	***	***	***	0.917	–

3-year time-dependent ROC					
^Index^AUC (95% CI)	^Index^AUC (95% CI)				
	^NLR^0.934 (0.867–0.925)	^GLR^0.842 (0.773–0.868)	^PLR^0.749 (0.675–0.789)	^LMR^0.645 (0.587–0.715)	^ALB^0.665 (0.616–0.737)
^NLR^0.934 (0.867–0.925)	–	*p* < 0.001	*p* < 0.001	*p* < 0.001	*p* < 0.001
^GLR^0.842 (0.773–0.868)	***	–	0.031	*p* < 0.001	*p* < 0.001
^PLR^0.749 (0.675–0.789)	***	0.031	–	*p* < 0.001	*p* < 0.001
^LMR^0.645 (0.587–0.715)	***	***	***	–	0.584
^ALB^0.665 (0.616–0.737)	***	***	***	0.584	–

5-year time-dependent ROC					
^Index^AUC (95% CI)	^Index^AUC (95% CI)				
	^NLR^0.996 (0.912–0.973)	^GLR^0.864 (0.792–0.889)	^PLR^0.737 (0.681–0.794)	^LMR^0.645 (0.583–0.714)	^ALB^0.665 (0.615–0.741)
^NLR^0.996 (0.912–0.973)	–	*p* < 0.001	*p* < 0.001	*p* < 0.001	*p* < 0.001
^GLR^0.864 (0.792–0.889)	***	–	0.006	*p* < 0.001	*p* < 0.001
^PLR^0.737 (0.681–0.794)	***	0.006	–	*p* < 0.001	*p* < 0.001
^LMR^0.645 (0.583–0.714)	***	***	***	–	0.509
^ALB^0.665 (0.615–0.741)	***	***	***	0.509	–

ROC, receiver operating characteristic; AUC, area under the curve; CI, confidence Interval; NLR, neutrophil to lymphocyte ratio; PLR, platelet to lymphocyte ratio; GLR, glucose to lymphocyte ratio; LMR, lymphocyte to monocyte ratio; ALB, albumin. *, *p* < 0.05; **, *p* < 0.01; ***, *p* < 0.001.

#### 3.3.3 Incremental effect of inflammation, glucose metabolism, and nutrition markers in predicting all-cause and cardiac mortality

**Figure 3:**
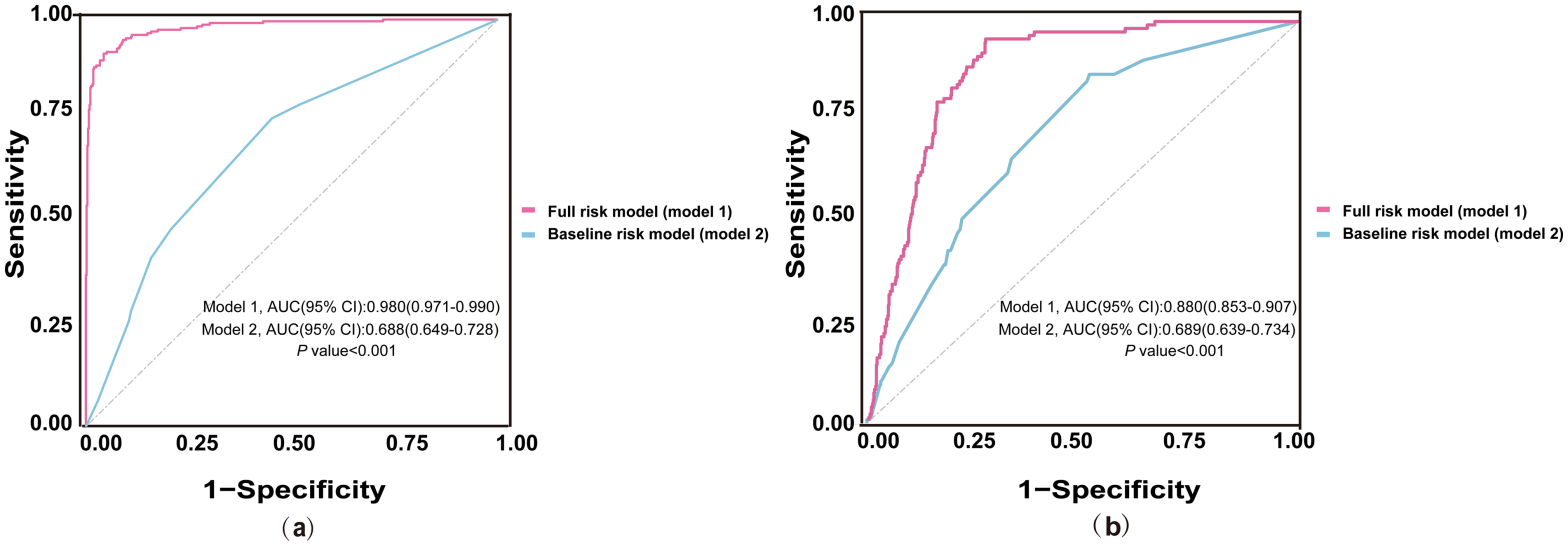
ROC curves of baseline and full risk models for predicting mortality. **(a)** ROC curves for predicting all-cause mortality; **(b)** ROC curves for predicting cardiac mortality. The blue line represents the baseline risk model including traditional risk factors (age, diabetes, hypertension, CHD). The red line represents the full risk model incorporating traditional risk factors and additional markers of nutrition (ALB), inflammation (NLR, PLR, LMR), and glucose metabolism (GLR). ROC, receiver operating characteristic; AUC, area under the curve; CI, Confidence Interval; NLR, neutrophil to lymphocyte ratio; CHD, coronary heart disease; PLR, platelet to lymphocyte ratio; GLR, glucose to lymphocyte ratio; LMR, lymphocyte to monocyte ratio; ALB, albumin.

In initial HD patients, ROC curves were generated to evaluate the predictive performance of two models for all-cause and cardiac mortality: a baseline risk model including traditional risk factors (age, diabetes, hypertension, and CHD) and a comprehensive risk model integrating traditional risk factors with inflammatory, glucose metabolism, and nutritional markers (Figure 3). The AUC values (95% CI) of the comprehensive model were 0.980 (0.971–0.990) for all-cause mortality and 0.880 (0.853–0.907) for cardiac mortality, both significantly higher than those of the baseline model [0.688 (0.649–0.728) and 0.689 (0.639–0.734), respectively] (all *p* < 0.001). As shown in Table 6, the C-index values of the comprehensive risk model for all-cause and cardiac mortality were 0.877 and 0.875, respectively, markedly exceeding those of the baseline model (0.648 and 0.680, respectively) (all *p* < 0.001). Furthermore, the category-free NRI values of the comprehensive risk model for predicting all-cause and cardiac mortality in the overall, deceased, and survivor cohorts were 0.793, 0.384 and 0.408, respectively. The IDI values were 0.356 and 0.216 for all-cause and cardiac mortality, respectively (all *p* < 0.001). These results indicated that incorporating inflammatory, glucose metabolism, and nutritional markers provided a significant incremental effect in predicting all-cause and cardiac mortality compared to the traditional risk model in patients initiating HD.

**Table 6:** Incremental effect of nutritional, inflammatory, and glucose metabolism markers in predicting all-cause and cardiac mortality.

Indicators		All-cause mortality		Cardiac mortality	
		Baseline risk model	Full risk model	Baseline risk model	Full risk model
C-index	Estimate (95% CI)	0.648 (0.612–0.683)	0.887 (0.872–0.903)	0.680 (0.631–0.929)	0.875 (0.852–0.897)
	Difference		−0.239		−0.195
	z-score		−14.495		−7.9548
	*p-*value	ref	<0.001	ref	<0.001
Category-free NRI	Total Estimate (95% CI)		0.793 (0.526–0.893)		0.793 (0.552–0.859)
	*p*-value	ref	<0.001	ref	<0.001
	Dead Estimate (95% CI)		0.384 (0.226–0.435)		0.384 (0.230–0.420)
	*p*-value	ref	<0.001	ref	<0.001
	Alive Estimate (95% CI)		0.408 (0.200–0.491)		0.408 (0.220–0.511)
	*p*-value	ref	<0.001	ref	<0.001
IDI	Estimate (95% CI)		0.356 (0.309–0.411)		0.216 (0.153–0.298)
	*p*-value	ref	<0.001	ref	<0.001

NRI, net reclassification index; IDI, integrated discrimination improvement; CI, confidence Interval; ref, reference.

#### 3.3.4 Nomograms incorporating inflammatory, glucose metabolism, and nutritional markers for predicting all-cause and cardiac mortality

**Figure 4:**
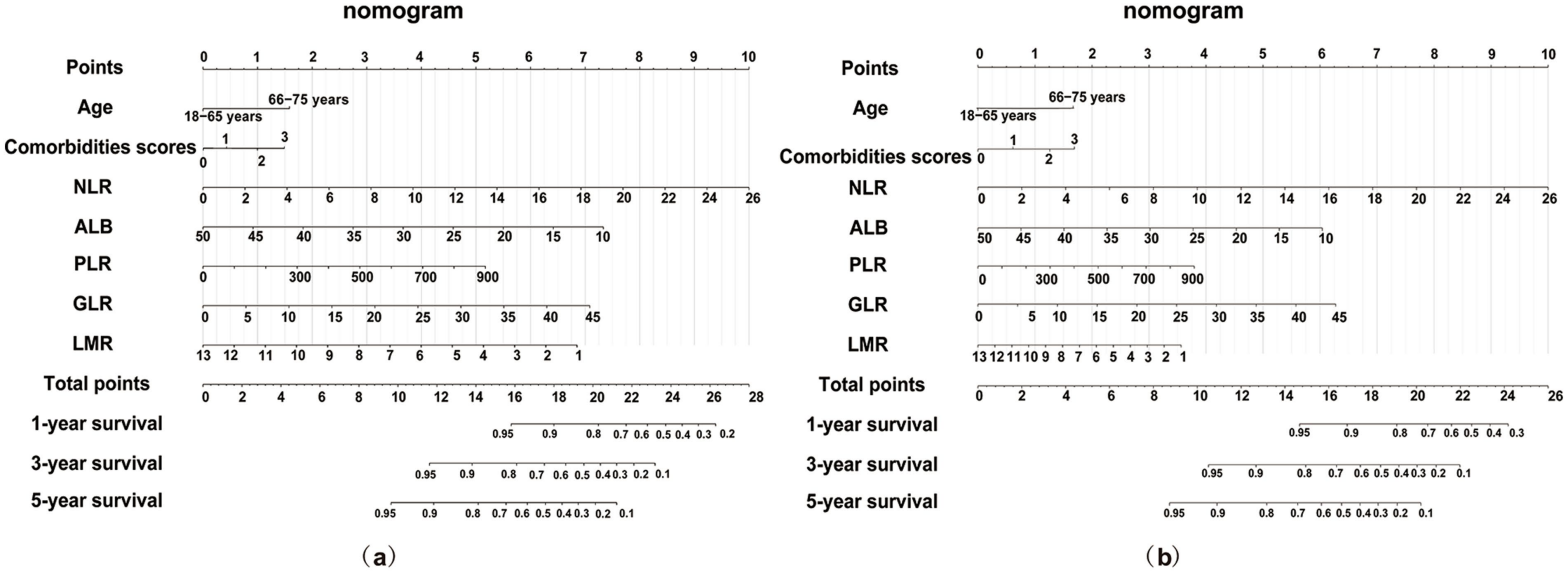
Nomogram for survival prediction based on the full risk model including age, comorbidities, NLR, GLR, PLR, LMR and ALB. **(a)** Nomogram for predicting all-cause mortality; **(b)** Nomogram for predicting cardiac mortality. NLR, neutrophil to lymphocyte ratio; PLR, platelet to lymphocyte ratio; GLR, glucose to lymphocyte ratio; LMR, lymphocyte to monocyte ratio; ALB, albumin.

Significant associations of NLR, PLR, GLR, LMR, and ALB with all-cause and cardiac mortality were observed in the training set (*n* = 557) (all *p* < 0.05) (Supplementary Tables 2, 3). Nomograms were constructed to predict 1-, 3-, and 5-year risks of all-cause (Figure 4a) and cardiac mortality (Figure 4b), incorporating traditional risk factors along with inflammatory, glucose metabolism, and nutritional markers. As an illustrative case, consider a 70-year-old patient with diabetes, hypertension and CHD. The baseline values include an NLR of 4, GLR of 25, PLR of 600, LMR of 6, and ALB of 25 g/L. According to the nomograms, the total scores for predicting all-cause and cardiac mortality are 17.25 and 11.25, respectively, corresponding to predicted survival probabilities at 1, 3, and 5 years of 92, 73, and 59% for all-cause mortality, and 90, 78, and 50% for cardiac mortality.

Validation metrics (C-index, time-dependent AUC, calibration, and DCA) confirming survival nomogram robustness are provided in Supplementary Figures 2–3.

## 4 Discussion

To our knowledge, this is the first study evaluating NLR, GLR, PLR, LMR, and ALB associations with all-cause and cardiac mortality in initial HD patients. Our multicenter analysis identified elevated NLR, GLR, PLR, and reduced LMR and ALB as independent risk factors for both outcomes. Each 1-unit increase in NLR and GLR raised all-cause mortality risk by 13 and 5%, and cardiac mortality by 13 and 5%, respectively. Every 10-unit PLR increase corresponded to a 2% risk increase. Conversely, each 1-unit decrease in LMR and ALB increased all-cause mortality by 18 and 6%, and cardiac mortality by 14 and 5%, respectively. NLR emerged as the strongest predictor for 1-, 3-, and 5-year mortality, outperforming the other markers. Routine NLR monitoring may enhance prognostic assessment. Incorporating these indices with traditional risk factors significantly improved mortality risk stratification, particularly long-term, highlighting their potential to optimize management for initial HD patients.

Patients undergoing HD frequently exhibit dysregulated adaptive immune responses, characterized by the release of pro-inflammatory cytokines and/or relative or absolute alterations in peripheral blood inflammatory cell counts. A typical hematologic profile includes elevated neutrophil, platelet, and monocyte counts, along with reduced lymphocyte counts—a pattern proven to correlate with poor clinical outcomes in dialysis populations (26–28). Chen et al. (17) confirmed elevated platelet counts in peritoneal dialysis patients with cardiovascular comorbidities, potentially indicating an “inflammatory-immune-thrombotic vicious cycle” (29, 30). Isolated white blood cell counts exhibit limited reliability due to inherent variability (31). Novel inflammatory indices (NLR, PLR, LMR) correlate strongly with traditional biomarkers (e.g., CRP, WBC, IL-6) and demonstrate prognostic value in the general population (32) and cancer patients (33). Among CKD/HD patients, Zhang et al. (34) linked elevated PLR to increased all-cause and cardiac mortality in Chinese HD patients, while NLR only associated with all-cause mortality. Conversely, Mayne et al. (19) found NLR—not PLR—predicted all-cause mortality in UK HD patients. Similarly, Chen et al. (35) identified NLR and LMR (not PLR) as predictors in US HD patients. Our multicenter cohort study demonstrated that NLR, PLR, and LMR were all independently associated with all-cause and cardiac mortality in initial HD patients. These findings suggest that NLR remains a robust predictor of all-cause mortality in HD patients, regardless of ethnic heterogeneity, while the prognostic value of PLR may differ by racial or regional factors. Notably, even when adjusting for NLR, PLR, and LMR simultaneously in multivariable models, NLR consistently demonstrated the strongest predictive performance for 1-, 3-, and 5-year mortality, followed by PLR and then LMR. This ranking is consistent with observations by Li et al. (14), who identified NLR as the most accurate predictor of 28-day all-cause mortality in sepsis patients with CHD, outperforming PLR and LMR. Given the consistent and reliable prognostic value of NLR across diverse populations and disease contexts, its translational utility for risk stratification in HD care warrants further attention.

Furthermore, in ESKD patients, relative or absolute insulin deficiency, resulting from reduced peripheral tissue insulin sensitivity, uremic toxin-mediated suppression of pancreatic *β*-cell receptor function, and impaired glucose utilization due to metabolic acidosis, contributes to elevated FBG and poor clinical outcomes (36, 37). The GLR, a novel biomarker, reflects systemic glucose dysregulation and chronic inflammation. Recent studies suggest that GLR offers superior prognostic value compared to its individual components (38). Moreover, Chen et al. (25) identified elevated serum GLR as an independent predictor of all-cause and cardiac mortality in PD patients. Yan et al. (39) reported that lowering GLR significantly reduced the risk of initial peritonitis episodes in PD populations. Our study is the first to confirm the robust predictive value of GLR for all-cause and cardiac mortality in HD patients, emphasizing its clinical significance. While GLR, NLR, PLR, and LMR each reflect disturbances in the metabolic-inflammatory axis, their biological interrelationships and disease-specific implications remain insufficiently characterized. We demonstrated that GLR, which positively correlated with NLR and PLR but inversely with LMR, maintained independent prognostic value for mortality outcomes in HD patients. The interplay between glucose metabolism and inflammation is mediated by complex, multi-organ regulatory networks. For instance, hyperglycemia may amplify inflammatory responses via oxidative stress, advanced glycation end products (AGEs) and endothelial injury, while inflammatory cytokines impair insulin receptor substrate (IRS) phosphorylation and suppress glucose transporter 4 (GLUT4) activity, exacerbating hyperglycemia (40) and insulin resistance (41). This bidirectional cycle likely contributes to poor outcomes, suggesting that effective HD management must target both sustained hyperglycemia and chronic inflammation.

Protein-Energy Wasting (PEW), a metabolic disorder characterized by the progressive loss of protein and energy reserves, is prevalent in HD patients (42). Hypoalbuminemia, a key diagnostic indicator of PEW, is strongly associated with increased mortality risk in dialysis populations (43), consistent with our findings in initial HD cohorts. Furthermore, we observed that ALB was inversely correlated with NLR, PLR, and GLR, but positively correlated with LMR. These associations may reflect the impact of inflammation on hepatic ALB synthesis, potentially mediated by the downregulation of ALB mRNA through pathways such as NF-κB activation (44). Although hypoalbuminemia remains independently associated with elevated mortality risk after mutual adjustment, its predictive power for all-cause and cardiac mortality is comparable to LMR but substantially lower than that of NLR, PLR, and GLR. These findings underscore the superior prognostic value of inflammatory and glucose-metabolic biomarkers over nutritional indicators alone in mortality risk stratification for HD patients.

Current evidence suggests that integrating multidimensional biomarkers offers a more comprehensive approach to risk stratification. Li et al. (44) demonstrated that while individual biomarkers such as NLR, LMR, PLR and the mSOFA score had limited predictive power for 28-day mortality in patients with septic coronary heart disease, combining these indicators with mSOFA significantly enhanced predictive accuracy. In our study, we were the first to incorporate nutritional (ALB), inflammatory (NLR, PLR, LMR), and glucose metabolism (GLR) biomarkers into traditional risk models that include age, diabetes, hypertension, and CHD. This integrative approach significantly improved the prediction of all-cause and cardiac mortality and demonstrated sustained effectiveness for long-term risk assessment. The death risk nomogram developed from the comprehensive model incorporates multi-dimensional parameters. This tool may assist clinicians in several key decision-making areas: First, the nomogram provides an intuitive and quantitative method to estimate individual patient risk, which can facilitate shared decision-making. For example, patients identified as high-risk may gain a clearer understanding of their prognosis, potentially motivating them to adhere to dietary recommendations, medication plans, and dialysis schedules. Conversely, those classified as low-risk can be reassured. Second, high-risk patients screened using the nomogram should receive intensified clinical monitoring, specifically through dietitian-led personalized interventions (to improve albumin and glucose levels) and tighter management of comorbidities such as diabetes and cardiovascular diseases. Third, this model helps identify a high-risk phenotype characterized by hyperglycemia–low serum albumin–inflammation. This population is ideal for future clinical trials targeting interventions designed to modify these risk factors, such as novel anti-inflammatory agents, specific nutritional supplements, or glycemic control strategies in non-diabetic HD patients.

However, this study still has several limitations. First, the retrospective design limits causal inference (as it is susceptible to unmeasured confounding); additionally, despite being multicenter, all centers are in China, limiting generalizability to diverse populations. To further validate our findings and establish causality, large-scale, prospective, multi-center studies involving diverse ethnic populations are warranted. This would help to enhance the generalizability of our results and account for potential genetic and socio-economic confounding factors. Second, although we adjusted for multiple confounding factors, the possibility of residual confounding remains. For instance, certain inflammatory markers—such as C-reactive protein and procalcitonin—were not included in the analysis due to severe data gaps resulting from inconsistent testing or documentation in clinical practice, and this may have influenced the results. Third, this study only analyzed baseline measurements of GLR and other laboratory parameters. Dynamic changes in these parameters during hemodialysis—such as fluctuations in albumin and glycemic variability—may hold greater prognostic significance, but were not explored in the current study. Future research should incorporate longitudinal data to evaluate the association between these time-dependent changes and patient outcomes. Fourth, unfortunately, this study did not include indicators for assessing dialysis adequacy, such as Kt/V urea (Kt/V). Considering that the study participants were patients initiating hemodialysis, who were in a phase of treatment adaptation and parameter adjustment, their dialysis regimens and metabolic status were not yet stable. During this stage, Kt/V values can be easily influenced by factors such as blood flow rate, dialysis duration, body weight estimation inaccuracies, and sampling timing, which may compromise the reliability and consistency of the results. Therefore, we believe that the absence of Kt/V has limited impact on the interpretation and reliability of the core findings of this study in the initial dialysis population. Despite these limitations, this is the most comprehensive study to date evaluating the relationship among nutritional status, inflammation, glucose metabolism, and mortality in patients undergoing initial HD. Future large-scale, multicenter, prospective cohort studies are warranted to validate these findings.

## 5 Conclusion

In conclusion, elevated NLR, GLR, and PLR, along with reduced LMR and ALB, were independent predictors for the all-cause and cardiac mortality in initial HD patients. Among these markers, NLR demonstrated the strongest predictive value, while GLR outperformed PLR in predicting long-term mortality. In addition, LMR and ALB showed comparatively weaker and similar predictive capacities. Importantly, a comprehensive risk model integrating these biomarkers with traditional risk factors significantly improved mortality risk stratification. Accordingly, we recommend the routine clinical use of a nomogram based on this integrated model to improve prognostic assessment and guide personalized management in patients initiating HD.

## Data Availability

The raw data supporting the conclusions of this article will be made available by the authors, without undue reservation.

## Footnotes

^1^version 4.3.1; http://www.r-project.org
