# Supplementary material for: Inflammation, glucose metabolism, and nutritional markers in relation to all-cause and cardiac mortality among initial hemodialysis patients: a multicenter cohort study
Source: Front Nutr. 2025 Nov 6;12:1660267. doi: 10.3389/fnut.2025.1660267 (PMC12631612; doi:10.3389/fnut.2025.1660267)
Supplement: Supplementary file 1 [file Table_1.docx]

**Supplementary material**

**Clinical covariates**

**Statistical analyses**

**Results**

**Supplementary Tables**

**Supplementary Figures**

**Clinical covariates：**

The following indices were calculated:

(1) Body mass index (BMI, kg/m²) = weight (kg) / height (m)².

(2) NLR = absolute neutrophil count / absolute lymphocyte count.

(3) PLR = absolute platelet count / absolute lymphocyte count.

(4) LMR = absolute lymphocyte count / absolute monocyte count.

(5) GLR = fasting glucose level / absolute lymphocyte count

**Statistical analyses：**

The construction and validation of the survival nomogram：

A survival nomogram was constructed using the training set based on the full-risk model, with comorbidity scores ranging from 0 to 3 points (1 point per comorbidity). The nomogram was designed to estimate 1-, 3-, and 5-year survival probabilities for both all-cause and cardiac mortality. Predictive accuracy was evaluated using the C-index, calibration curves, time-dependent ROC analysis, and decision curve analysis (DCA).

**Results:**

- Correlation analyses of inflammation, glucose metabolism, and nutrition markers:

As shown in Supplementary Figure 1, the nutritional marker ALB was negatively correlated with the inflammatory markers NLR, PLR and the glucose metabolism marker GLR, but positively correlated with LMR (all *P* < 0.05). In turn, GLR showed positive correlations with NLR and PLR, and negative correlations with LMR and ALB (all *P* < 0.05).

- Validation metrics (C-index, time-dependent AUC, calibration, and DCA) confirming survival nomogram robustness:

In the training and internal validation sets, the C-index values for the all-cause mortality were 0.885 (0.865-0.905) and 0.871 (0.842-0.900), respectively, while those for the cardiac mortality were 0.895 (0.868–0.922) and 0.902 (0.863–0.941), respectively, indicating strong discriminatory performance. Calibration curve analysis (Supplementary Figure 2a-d) demonstrated high consistency between the predicted probabilities and the actual observed outcomes at 1, 3, and 5 years for both all-cause and cardiac survival models. The AUC values for the all-cause survival model at 1, 3, and 5 years were 0.848, 0.933, 0.945 in the training set, and 0.892, 0.915, 0.966 in the validation set (Supplementary Figure 2e-f). Corresponding AUC values for the cardiac mortality model were 0.831, 0.978, 0.989 in the training set, and 0.906, 0.947, 0.978 in the validation set (Supplementary Figure 2g-h), confirming the models' robustness and predictive accuracy. DCA (Supplementary Figure 3a-l) further revealed that the net clinical benefit of the all-cause and cardiac mortality models was evident within the 10%–25% threshold range at 1 year, expanding to 10%–80% at 5 years, suggesting that long-term risk predictions can enhance the identification of individuals who may benefit from timely clinical interventions.

**Supplementary Tables:**

**Supplementary Table 1** Univariate and multivariate Cox regression analyses for all-cause mortality in training set (n=557)

| Characteristics | Univariate Cox regression analyses | |  | Multivariate Cox regression analyses | |
| --- | --- | --- | --- | --- | --- |
|  | HR ( 95% CI ) | *P*-value |  | HR ( 95% CI ) | *P*-value |
| Sex ( Male ) | 1.021 (0.751, 1.378) | 0.923 |  |  |  |
| Age ( 66-75 years ) | 2.724 (2.011, 3.695) | **< 0.001** |  | 1.462 (1.041, 2.022) | **0.028** |
| BMI, kg/m² | 0.964 (0.933, 1.010) | 0.108 |  |  |  |
| Smoking history | 1.235 (0.878, 1.737) | 0.212 |  |  |  |
| Diabetes | 1.853 (1.371, 2.501) | **< 0.001** |  |  |  |
| Hypertension | 0.863 (0.511, 1.438) | 0.563 |  |  |  |
| CHD | 1.619 (1.110, 2.364) | **0.012** |  |  |  |
| Hb, g/L | 1.010 (1.001, 1.013) | 0.188 |  |  |  |
| NLR | 1.234 (1.201, 1.257) | **< 0.001** |  | 1.157 (1.132, 1.189) | **< 0.001** |
| PLR | 1.004 (1.003, 1.009) | **< 0.001** |  | 1.002 (1.001, 1.003) | **0.002** |
| GLR | 1.110 (1.090, 1.118) | **< 0.001** |  | 1.053 (1.041, 1.065) | **< 0.001** |
| LMR | 0.621 (0.554, 0.713) | **< 0.001** |  | 0.854 (0.752, 0.955) | **0.008** |
| ALB, g/L | 0.889 (0.879, 0.933) | **< 0.001** |  | 0.943 (0.912, 0.974) | **< 0.001** |
| β-blocker | 0.643 (0.481, 0.862) | **0.003** |  |  |  |
| ACEI/ARB | 1.202 (0.867, 1.664) | 0.276 |  |  |  |
| Lipid lowing agents | 1.542 (1.111, 2.132) | **0.009** |  |  |  |
| SCr/CysC | 1.003 (1.001, 1.013) | 0.084 |  |  |  |

Abbreviations: HR, hazard ratio; CI, confidence Interval; BMI, body mass index; CHD, coronary heart disease; Hb, hemoglobin; NLR, neutrophil to lymphocyte ratio; PLR, platelet to lymphocyte ratio; GLR, glucose to lymphocyte ratio; LMR, lymphocyte to monocyte ratio; ALB, albumin; ACEI, angiotensin-converting enzyme inhibitor; ARB, angiotensin receptor blocker; SCr/CysC, ratio of serum creatinine to cystatin C.

**Supplementary Table 2** Univariate and multivariate Cox regression analyses for cardiac mortality in training set (n=557)

| Characteristics | Univariate Cox regression analyses | |  | Multivariate Cox regression analyses | |
| --- | --- | --- | --- | --- | --- |
|  | HR ( 95% CI ) | *P*-value |  | HR ( 95% CI ) | *P*-value |
| Sex ( Male ) | 1.172 (0.744, 1,830) | 0.505 |  |  |  |
| Age ( 66-75 years ) | 2.644 (1.709, 4.105) | **< 0.001** |  | 1.643 (1.052, 2.544) | **0.028** |
| BMI, kg/m² | 0.995 (0.951, 1.053) | 0.992 |  |  |  |
| Smoking history | 1.133 (0.695, 1.857) | 0.625 |  |  |  |
| Diabetes | 2.276 (1.473, 3.541) | **< 0.001** |  |  |  |
| Hypertension | 0.827 (0.406, 1.704) | 0.600 |  |  |  |
| CHD | 2.683 (1.672, 4.299) | **< 0.001** |  | 1.711 (1.062, 2.756) | **0.028** |
| Hb, g/L | 1.010 (1.004, 1.024) | **0.042** |  |  |  |
| NLR | 1.245 (1.201, 1.284) | **< 0.001** |  | 1.154 (1.113, 1.201) | **< 0.001** |
| PLR | 1.002 (1.003, 1.005) | **< 0.001** |  | 1.002 (1.001, 1.004) | **0.041** |
| GLR | 1.105 (1.010, 1.128) | **< 0.001** |  | 1.041 (1.013, 1.065) | **0.002** |
| LMR | 0.687 (0.588, 0.825) | **< 0.001** |  | 0.887 (0.763, 0.952) | **0.016** |
| ALB, g/L | 0.922 (0.878, 0.952) | **< 0.001** |  | 0.943 (0.906, 0.981) | **0.002** |
| β-blocker | 0.880 (0.573, 1.362) | 0.571 |  |  |  |
| ACEI/ARB | 1.166 (0.724, 1.863) | 0.546 |  |  |  |
| Lipid lowing agents | 1.911 (1.222, 3.001) | **0.005** |  |  |  |
| SCr/CysC | 1.002 (1.001, 1.010) | 0.056 |  |  |  |

Abbreviations: HR, hazard ratio; CI, confidence Interval; BMI, body mass index; CHD, coronary heart disease; Hb, hemoglobin; NLR, neutrophil to lymphocyte ratio; PLR, platelet to lymphocyte ratio; GLR, glucose to lymphocyte ratio; LMR, lymphocyte to monocyte ratio; ALB, albumin; ACEI, angiotensin-converting enzyme inhibitor; ARB, angiotensin receptor blocker; SCr/CysC, ratio of serum creatinine to cystatin C.


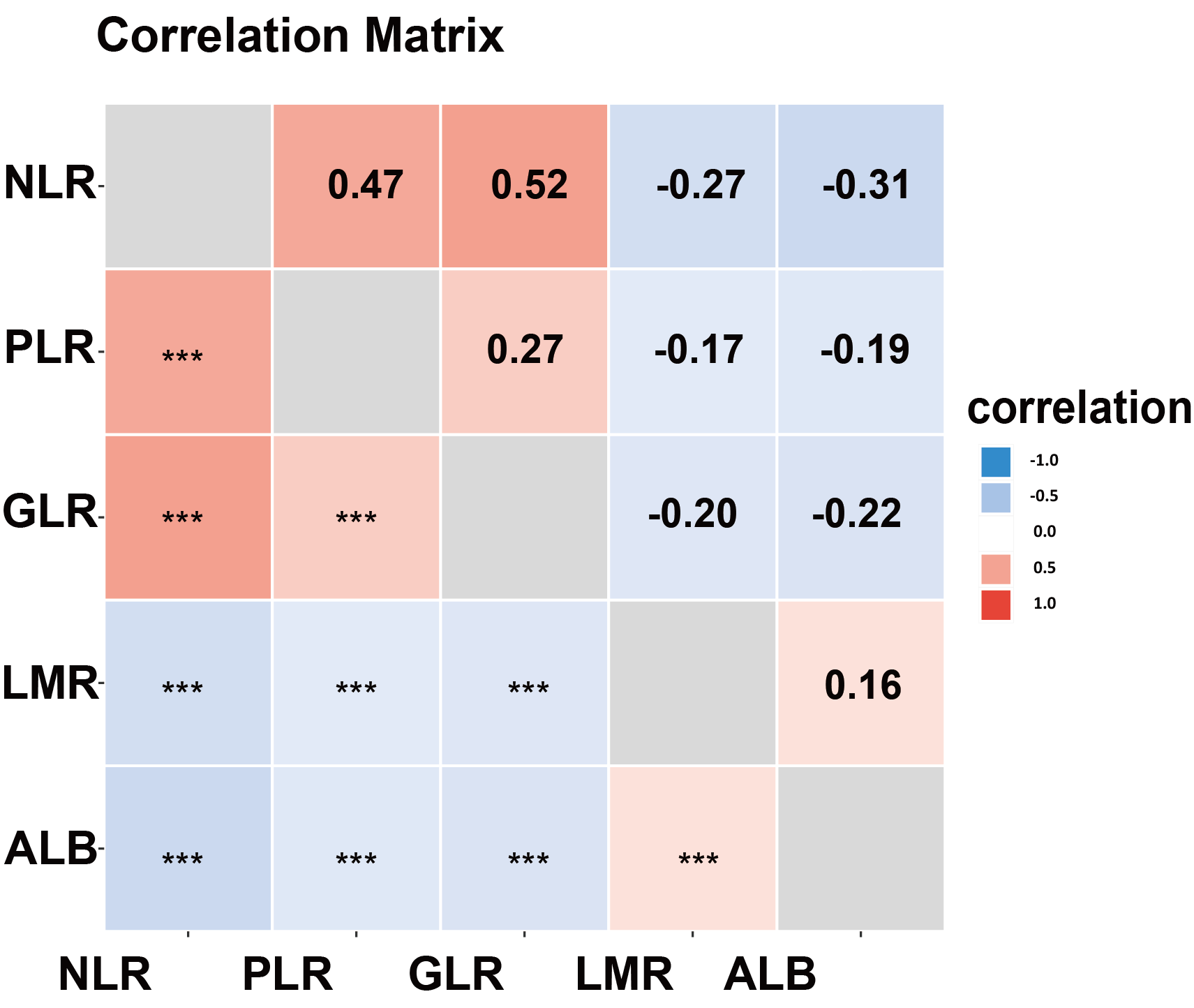


**Supplementary Figure 1** Correlation analysis of nutritional, inflammatory and glucose metabolism markers. The correlation coefficient ranges from -1 to +1, where +1 indicates a perfect positive correlation and -1 represents a perfect negative correlation. Red denotes positive correlations, white indicates values near zero, and blue signifies negative correlations. Darker shades correspond to stronger correlations. Abbreviations: NLR, neutrophil to lymphocyte ratio; PLR, platelet to lymphocyte ratio; GLR, glucose to lymphocyte ratio; LMR, lymphocyte to monocyte ratio; ALB, albumin. *, *P* < 0.05; **, *P* < 0.01; ***, *P* < 0.001.


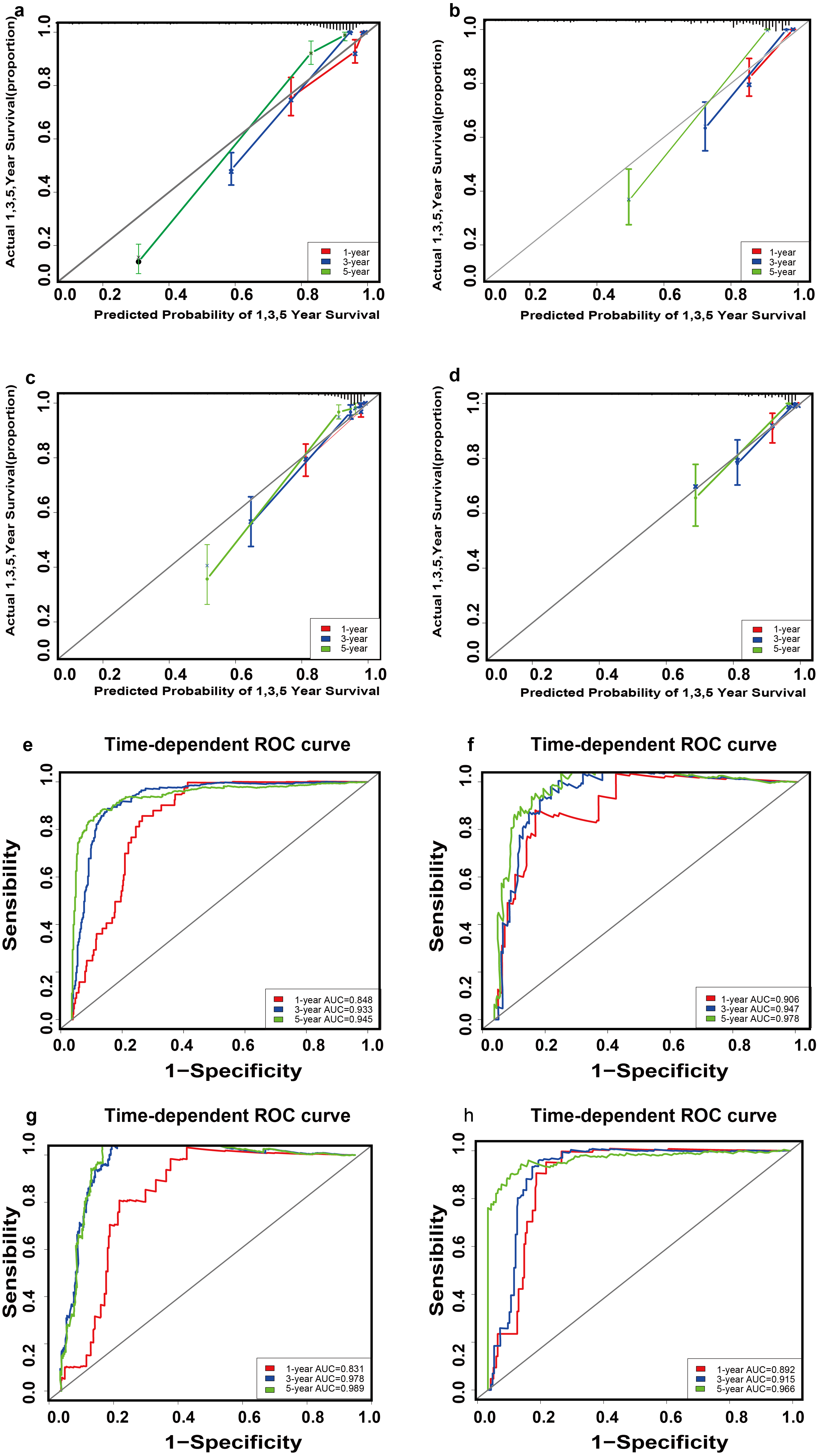


**Supplementary Figure 2** Evaluation of survival nomograms in patients initiating hemodialysis. (a-b) Calibration curves for 1,3,5-year overall survival rate prediction in the training (a) and internal verification set (b). (c-d) Calibration curves for 1,3,5-year non-cardiac mortality-free survival rate prediction in the training (c) and internal verification set (d). (e-f) Time-dependent ROC curves for 1,3,5-year overall survival rate prediction in the training (e) and internal validation set (f). (g-h) Time-dependent ROC curves for 1,3,5-year non-cardiac mortality-free survival rate prediction in the training (g) and internal validation set (h). Abbreviations: ROC, receiver operating characteristic; AUC, area under the curve; CI, Confidence Interval; NLR, neutrophil to lymphocyte ratio; PLR, platelet to lymphocyte ratio; GLR, glucose to lymphocyte ratio; LMR, lymphocyte to monocyte ratio; ALB, albumin.


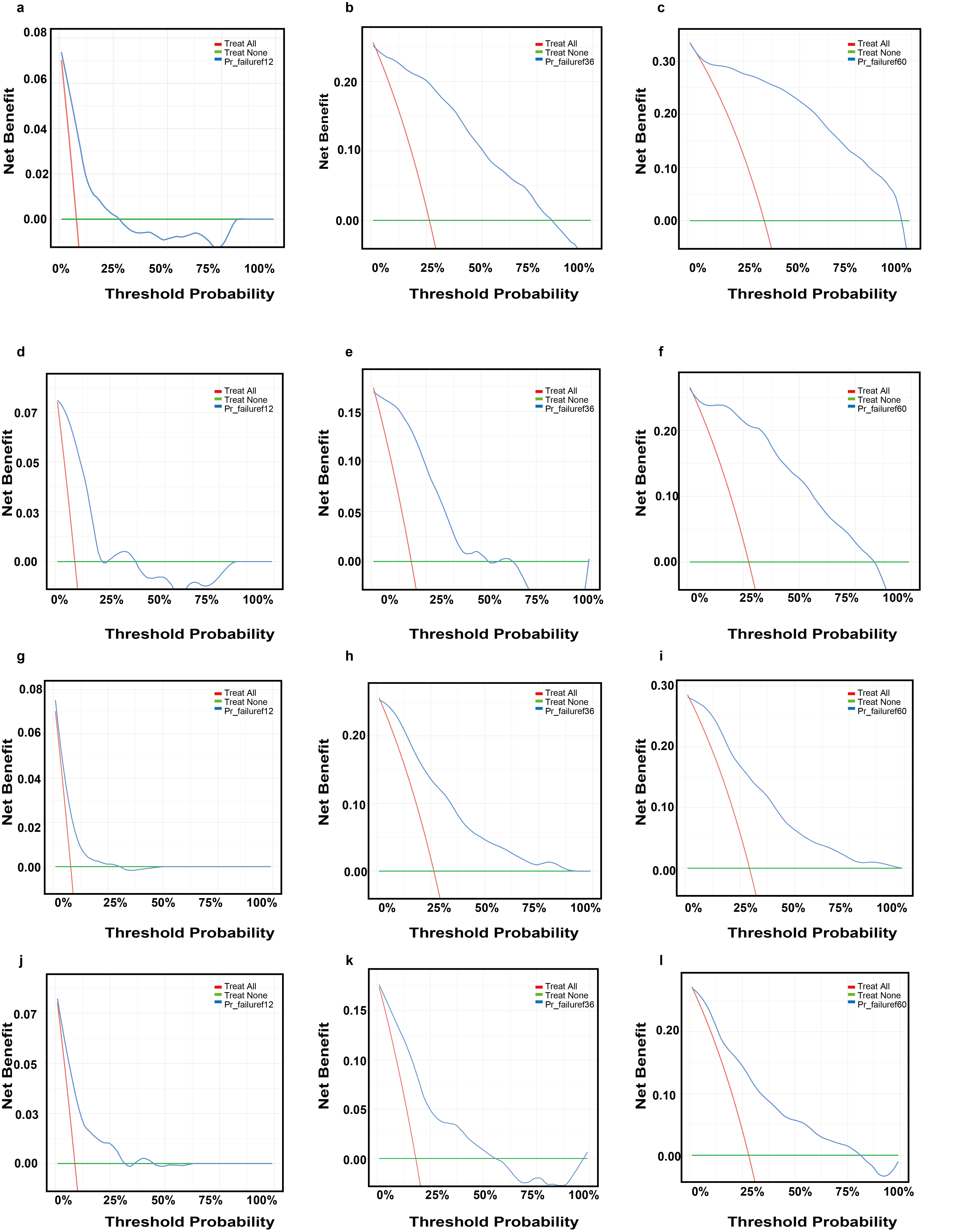


**Supplementary Figure 3** DCA for the proposed nomograms. DCAs for the proposed nomogram model to predict 1,3,5-year overall survival rate in the training (a-c) and internal validation set (d-f). DCAs for the proposed nomogram model to predict 1,3,5-year non-cardiac mortality-free survival rate in the training (g-i) and internal validation set (j-l). DCA: decision curve analyses.

STROBE Statement—Checklist of items that should be included in reports of ***cohort studies***

|  | **Item No** | **Recommendation** | **Page No** |
| --- | --- | --- | --- |
| **Title and abstract** | 1 | (*a*) Indicate the study’s design with a commonly used term in the title or the abstract |  |
|  |  | (*b*) Provide in the abstract an informative and balanced summary of what was done and what was found | 1,3 |
| **Introduction** | | | |
| Background/rationale | 2 | Explain the scientific background and rationale for the investigation being reported | 4 |
| Objectives | 3 | State specific objectives, including any prespecified hypotheses | 4 |
| **Methods** | | | |
| Study design | 4 | Present key elements of study design early in the paper | 5 |
| Setting | 5 | Describe the setting, locations, and relevant dates, including periods of recruitment, exposure, follow-up, and data collection | 5 |
| Participants | 6 | (*a*) Give the eligibility criteria, and the sources and methods of selection of participants. Describe methods of follow-up | 5 |
|  |  | (*b*) For matched studies, give matching criteria and number of exposed and unexposed |  |
| Variables | 7 | Clearly define all outcomes, exposures, predictors, potential confounders, and effect modifiers. Give diagnostic criteria, if applicable | 5,6, Supplementary material |
| Data sources/ measurement | 8* | For each variable of interest, give sources of data and details of methods of assessment (measurement). Describe comparability of assessment methods if there is more than one group | 5, Supplementary material |
| Bias | 9 | Describe any efforts to address potential sources of bias | 5 |
| Study size | 10 | Explain how the study size was arrived at | 4 |
| Quantitative variables | 11 | Explain how quantitative variables were handled in the analyses. If applicable, describe which groupings were chosen and why | 5, |
| Statistical methods | 12 | (*a*) Describe all statistical methods, including those used to control for confounding | 6,7, Supplementary material |
|  |  | (*b*) Describe any methods used to examine subgroups and interactions |  |
|  |  | (*c*) Explain how missing data were addressed |  |
|  |  | (*d*) If applicable, explain how loss to follow-up was addressed |  |
|  |  | (*e*) Describe any sensitivity analyses |  |
| **Results** | | |  |
| Participants | 13* | (a) Report numbers of individuals at each stage of study—eg numbers potentially eligible, examined for eligibility, confirmed eligible, included in the study, completing follow-up, and analysed | 7 |
|  |  | (b) Give reasons for non-participation at each stage |  |
|  |  | (c) Consider use of a flow diagram |  |
| Descriptive data | 14* | (a) Give characteristics of study participants (eg demographic, clinical, social) and information on exposures and potential confounders | 7, 8 |
|  |  | (b) Indicate number of participants with missing data for each variable of interest |  |
|  |  | (c) Summarise follow-up time (eg, average and total amount) |  |
| Outcome data | 15* | Report numbers of outcome events or summary measures over time | 7 |

| Main results | 16 | (*a*) Give unadjusted estimates and, if applicable, confounder-adjusted estimates and their precision (eg, 95% confidence interval). Make clear which confounders were adjusted for and why they were included | 8-9 |
| --- | --- | --- | --- |
|  |  | (*b*) Report category boundaries when continuous variables were categorized |  |
|  |  | (*c*) If relevant, consider translating estimates of relative risk into absolute risk for a meaningful time period |  |
| Other analyses | 17 | Report other analyses done—eg analyses of subgroups and interactions, and sensitivity analyses | 8,10, Supplementary material |
| **Discussion** | | | |
| Key results | 18 | Summarise key results with reference to study objectives | 10 |
| Limitations | 19 | Discuss limitations of the study, taking into account sources of potential bias or imprecision. Discuss both direction and magnitude of any potential bias | 13 |
| Interpretation | 20 | Give a cautious overall interpretation of results considering objectives, limitations, multiplicity of analyses, results from similar studies, and other relevant evidence | 10-13 |
| Generalisability | 21 | Discuss the generalisability (external validity) of the study results | 12, 13 |
| **Other information** | | | |
| Funding | 22 | Give the source of funding and the role of the funders for the present study and, if applicable, for the original study on which the present article is based | 15 |

*Give information separately for exposed and unexposed groups.

**Note:** An Explanation and Elaboration article discusses each checklist item and gives methodological background and published examples of transparent reporting. The STROBE checklist is best used in conjunction with this article (freely available on the Web sites of PLoS Medicine at http://www.plosmedicine.org/, Annals of Internal Medicine at http://www.annals.org/, and Epidemiology at http://www.epidem.com/). Information on the STROBE Initiative is available at http://www.strobe-statement.org.
